# P2X_7_ Receptors in Neurological and Cardiovascular Disorders

**DOI:** 10.1155/2009/861324

**Published:** 2009-06-24

**Authors:** Stephen D. Skaper, Patrizia Debetto, Pietro Giusti

**Affiliations:** Department of Pharmacology and Anesthesiology, University of Padova, Largo “E. Meneghetti” 2, 35131 Padova, Italy

## Abstract

P2X receptors are ATP-gated cation channels that mediate fast excitatory transmission in diverse regions of the brain and spinal cord. Several P2X receptor subtypes, including P2X_7_, have the unusual property of changing their ion selectivity during prolonged exposure to ATP, which results in a channel pore permeable to molecules as large as 900 daltons. The P2X_7_ receptor was originally described in cells of hematopoietic origin, and mediates the influx of Ca^2+^ and Na^+^ and Ca^2+^ and Na^+^ ions as well as the release of proinflammatory cytokines. P2X_7_ receptors may affect neuronal cell death through their ability to regulate the processing and release of interleukin-1*β*, a key mediator in neurodegeneration, chronic inflammation, and chronic pain. Activation of P2X_7_, a key mediator in neurodegeneration, chronic inflammation, and chronic pain. Activation of P2X_7_ receptors provides an inflammatory stimulus, and P2X_7_ receptor-deficient mice have substantially attenuated inflammatory responses, including models of neuropathic and chronic inflammatory pain. Moreover, P2X_7_ receptor activity, by regulating the release of proinflammatory cytokines, may be involved in the pathophysiology of depression. Apoptotic cell death occurs in a number of vascular diseases, including atherosclerosis, restenosis, and hypertension, and may be linked to the release of ATP from endothelial cells, P2X_7_ receptor activation, proinflammatory cytokine production, and endothelial cell apoptosis. In this context, the P2X_7_ receptor may be viewed as a gateway of communication between the nervous, immune, and cardiovascular systems.

## 1. Introduction

The role of extracellular ATP and purinoceptors in cytokine regulation and neurological disorders is the focus of a rapidly expanding area of research. ATP can act as a neurotransmitter, while the presence of the purinergic receptor subclass P2X_7_ on immune cells suggests that it also regulates immune function and inflammatory responses. In addition, activation of this receptor has dramatic cytotoxic properties which, together with its ability to regulate cytokine production and release, propose that it can act as an important regulator of cell death in response to pathological insults in both nervous and other (e.g., cardiovascular) tissues.

**Figure 1:**
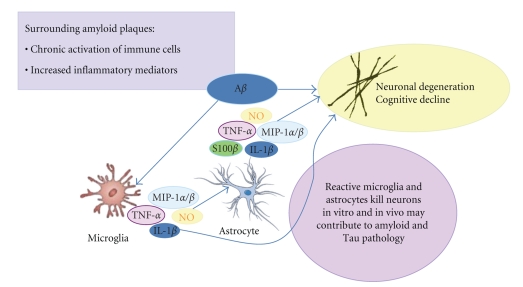
The inflammatory cycle and neurodegeneration: Alzheimer's disease as a case in point. In Alzheimer's disease, the neuroinflammatory cycle is characterized by sustained activation of microglia and astrocytes in response to activating stimuli, in particular, amyloid beta (A*β*). Glia proinflammatory responses activated by A*β* include induction of cytokines (TNF-*α*, IL-1*β*, S100*β*), chemokines (macrophage inflammatory proteins-1*α, β*: MIP-1*α*, MIP-1*β*), and oxidative stress-related molecules (nitric oxide, NO), which can cause neuronal cell dysfunction and/or death and can further propagate the inflammatory response.

Neurodegeneration is the underlying basis of many disorders including cerebral ischemia, brain trauma, multiple sclerosis, Parkinson's, Alzheimer's, and Huntington's diseases. Neuroinflammation in disorders such as Alzheimer's disease (AD) has previously been viewed as an epiphenomenon, with inflammation occurring when damaged neurons provoke an activation response from glia. Accumulating evidence now challenges this perspective and points to a more active role of neuroinflammation in pathophysiology onset and progression. In the central nervous system (CNS), glial cells (microglia, astroglia, and oligodendroglia) not only serve supportive and nutritive roles for neurons but also in the healthy brain often respond to stress and insults by transiently upregulating inflammatory processes. These processes are kept in check by other endogenous anti-inflammatory and neuroprotective responses that return the brain to homeostasis. Otherwise “normal” glial functions can sometimes result in a more severe and chronic neuroinflammatory cycle that actually promotes or propagates neurodegenerative disease [1]. The delicate balance in this homeostasis can be disturbed, resulting in disease or exacerbation of initiating factors that result in disease (i.e., the neuroinflammation hypothesis) (Figure 1) [2].

Clinical evidence in support of neuroinflammation as a pharmacological target for chronic neurodegenerative diseases, such as AD, comes from epidemiological and genetic linkage data. For example, long-term use of nonsteroidal anti-inflammatory drugs is correlated with a protective effect against AD [3], and certain polymorphisms in the genes for proinflammation mediators are associated with increased risk [4]. Postmortem brain tissue from patients with AD has expressed an array of inflammatory mediators, including cytokines and chemokines in those regions most affected in the disorder [5]. Neuroinflammation has been documented also in the affected brain regions of individuals suffering from Parkinson's disease, multiple sclerosis, human immunodeficiency virus type 1-associated dementia, and various prion diseases [6–9], as well as cerebral ischemia [10], spinal cord injury [11], and traumatic brain injury [12]. The ability of P2X_7_ receptor activation to regulate cytokine production and cellular vitality has implications for other neurological disorders, for example, pain and depression, as well as within the cardiovascular system. Collectively these observations propose that excessive P2X_7_ receptor activation on both glia and vascular cells constitutes a viable target for the discovery and development of novel disease therapeutics. This review will discuss the current biology and cellular signaling pathways of P2X_7_ receptor function, as well as insights into the role for this receptor in neurological/psychiatric and cardiovascular diseases, and the therapeutic potential of P2X_7_ receptor antagonism.

## 2. P2X_7_ Receptor Biology

Virtually all cell types express plasma membrane receptors for extracellular nucleotides, named P2 receptors. Presently, 15 members have been cloned and are classified into two subfamilies: the G protein-coupled P2Y receptors and P2X receptors [13, 14]. P2X receptors function as ATP-gated nonselective cationic channels permeable to Na^+^, K^+^, and Ca^2+^ [15]. The ability of P2X receptors to act as direct conduits for Ca^2+^ influx or indirect activators of voltage-gated Ca^2+^ channels underlies their multiple roles in Ca^2+^-based signaling responses in those tissues. The channels are oligomeric complexes composed of protein subunits encoded by seven different P2X receptor genes (*P*2*X*_1_ through *P*2*X*_7_) expressed in mammalian and other vertebrate genomes. The minimum stoichiometric conformation of the P2X_7_ receptor channel appears to be a trimer [13, 16]. Whether pore formation results from intrinsic dilation of the channel [13] or P2X_7_ receptor-mediated downstream signaling remains to be fully resolved.

**Figure 2:**
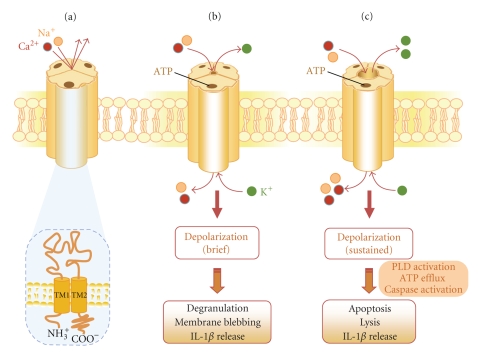
Structure and signaling functions of the P2X_7_ receptor. (a) Each functional P2X_7_ receptor is a trimer [18], with the three protein subunits arranged around a cation-permeable channel pore. The subunits all share a common topology, possessing two plasma membrane spanning domains (TM1 and TM2), a large extracellular loop with the ATP binding site, and containing 10 similarly spaced cysteines and glycosylation sites, and intracellular carboxyl and amino termini. (b) Brief ATP activation (<10 seconds) of the P2X_7_ receptor results in rapid and reversible channel opening that is permeable to Na^+^, K^+^, and Ca^2+^. Acute receptor activation also triggers a series of cellular responses, such as depolarization, degranulation, and membrane blebbing, along with signaling cascades (see Figure 3 for further details). (c) Continued stimulation results in the formation of a larger plasma membrane pore, which facilitates the uptake of cationic molecules up to 900 Da (including ethidium bromide, which is frequently used as a tool to measure channel permeability, based on its property of generating a fluorescent signal upon DNA binding). Further activation of the receptor in some cell types results in the induction of apoptosis/cell lysis. ATP-induced increase in IL-1*β* release is mediated mainly through the activation of IL-1*β* converting enzyme (also known as caspase-1). Activation of the P2X_7_ receptor triggers the efflux of K^+^ from cells which in turn activates IL-1 converting enzyme, leading to cleavage of pro-IL-1*β* to mature IL-1*β* and release from the cell.

All functional P2X receptor subtypes display a very high selectivity for ATP over other physiological nucleotides [16]. The P2X_7_ receptor is unusual among the P2X receptor family in that sustained activation by extracellular ATP causes the formation of a reversible plasma membrane pore permeable to hydrophilic solutes up to 900 Da [13]. This property is likely due to the receptor's extended carboxy terminal domain [17]. The P2X_7_ receptor activates a diverse range of cellular responses including phospholipase A_2_, phospholipase D, mitogen-activated protein kinase (MAPK), and nuclear factor-kappa B (NF-*κ*B) (Figure 2) [13].

P2X_7_ receptors are selectively expressed on cells of hematopoietic lineage including mast cells, erythrocytes, monocytes, peripheral macrophages, dendritic cells, T- and B-lymphocytes, and epidermal Langerhans cells [13]. Within the CNS, functional P2X_7_ receptors are localized on microglia and Schwann cells as well as on astrocytes [19, 20]. The existence of functional P2X_7_ receptors on peripheral or central neurons remains controversial owing to the poor selectivity of both antibodies and ligands targeting the rat P2X_7_ receptor [21]. In rat peripheral sensory ganglia (dorsal root), P2X_7_ receptors appear to be selectively localized on glial cells, but not neurons [22]. The best characterized activity of the P2X_7_ receptor is its role in interleukin-1*β* (IL-1*β*) release from macrophages and microglia that have been primed with substances such as bacterial endotoxin (lipopolysaccharide, LPS) [23]. Protracted activation of P2X_7_ receptors in some cell types results in the induction of apoptosis [13, 24]. However, the physiological significance of this “highly stimulated” state of the P2X_7_ receptor is unclear.

The only known physiological activator of the P2X_7_ receptor is ATP. It is remarkable that activation of the P2X_7_ receptor requires near millimolar concentrations of ATP (EC_50_≅300 *μ*M). Since the cytoplasmic ATP concentration is in the millimolar range, acute cell injury or death will cause massive ATP release into the extracellular milieu. Indeed, activated immune cells [25], macrophages [26], microglia [27], platelets [28], and dying cells may release high concentrations of nucleotide di- and tri-phosphates into the extracellular space [29]. Extracellular ATP concentrations increase significantly under inflammatory conditions in vivo [30] and in response to tissue trauma [31], suggesting that ATP levels sufficient to activate the P2X_7_ receptor may be reached in the pericellular space [28]. In addition, proinflammatory cytokines and bacterial products up-regulate P2X_7_ receptor expression and increase its sensitivity to extracellular ATP [32, 33].

Deletion of *P*2*X*_7_ abolishes the ability of extracellular ATP to induce IL-1*β* release from isolated macrophages [34]. P2X_7_ receptor-deficient mice are protected against symptom development and cartilage destruction in anticollagen antibody-induced arthritis [35]. Disruption of the P2X_7_ receptor gene abolishes chronic inflammatory and neuropathic pain [36], and may play a role in the pathophysiology of AD [37]. Recent studies suggest a link between the P2X_7_ receptor gene and both neuropsychiatric [38] and cardiovascular diseases [39]. These topics will be covered in detail in later sections.

## 3. P2**X**_7_ Receptor Signaling

**Figure 3:**
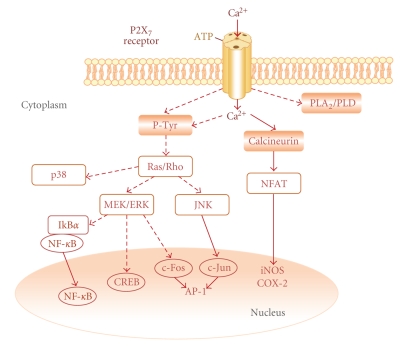
Schematic depiction of the signal transduction events occurring in microglia following P2X_7_ receptor activation. Extracellular calcium influx triggered by activation of ionotropic P2X_7_ receptors leads to activation of calcineurin and dephosphorylation/activation of NFAT (nuclear factor of activated T cells). P2X_7_ receptor activation also results in activation of phospholipases A_2_ and D (PLA_2_, PLD), as well as tyrosine phosphorylation (P-Tyr) and activation of mitogen-activated protein kinase (MAPK) pathway proteins (MAPK kinase, MEK; extracellular signal-regulated kinase, ERK). The latter can then influence the activity of transcription factors like NF-*κ*B (nuclear factor-*κ*B), CREB (cyclic AMP response element (CRE)-binding protein), and AP-1 (activator protein-1) which upregulate expression of pro-inflammatory genes, such as cyclooxgenase-2 (COX-2) and inducible nitric oxide synthase (iNOS). Activation of P2X_7_ receptors also leads to p38 MAPK activation with consequent phosphorylation/activation of CREB. Broken lines indicate multistep pathways.

In macrophages/monocytes, P2X_7_ receptor stimulation rapidly activates c-Jun N-terminal kinases 1 and 2 (JNK-1/2) [40], extracellular signal-regulated kinase (ERK-1/2), and p38 MAPK [41]. The P2X_7_ receptor agonist 2′, 3′-*O*-(4-benzoyl-benzoyl)ATP (BzATP) activates the same pathways in mouse N9 microglia [42], and increases the nuclear translocation of NF-*κ*B in mouse BV-2 microglia [42]. Dephosphorylation of NFAT (nuclear factor of activated T cells) by calcineurin exposes a nuclear localization sequence, permitting nuclear translocation and transcriptional activation [43]. In N9 cells, ATP activates NFAT via the P2X_7_ receptors in a calcineurin-dependent fashion [44]. The cyclic AMP response element- (CRE-) binding protein (CREB), a member of the ATF-1 (activating transcription factor 1) family of transcription factors, is involved in cytokine gene regulation [45]. P2X_7_ receptor-dependent induction of CREB and ATF-1 phosphorylation occurs in BV-2 cells via an MAPK kinase (MEK)/ERK-dependent pathway [42]. Activation of activator protein-1 (AP-1) is another transcription factor associated with regulation of inflammatory genes [46]. Multiple members of the c-Fos and c-Jun families dimerize to form AP-1. In serum-starved Jurkat T-cells, activation of P2X_7_ receptors induced AP-1 DNA binding activity as a result of increased c-Jun and c-Fos expression [47]. ATP treatment also increased the phosphorylation of ERK-1/2 and JNK-1/2, but not p38 MAPK, providing a potential mechanism for these effects (Figure 3).

Stimulation of P2X_7_ receptors increases protein tyrosine phosphorylation [48, 49] ultimately leading to MAPK pathway activation. Many events downstream of P2X_7_ receptor activation are dependent upon extracellular calcium influx [13, 44], and activation of MAPK pathways by P2X_7_ receptors may involve calcium signaling. In RAW 264.7 macrophages, the calcium-dependent kinase Pyk2, which facilitates Ras activation, is tyrosine phosphorylated in response to treatment with BzATP [48, 50], potentially linking calcium fluxes, Ras activation, and MAPK pathways with P2X_7_ receptors. P2X_7_ receptors also induce the activation of other small molecular weight G-proteins. For example, the Rho/p38 pathway may be involved in the shedding of IL-1*β*-containing vesicles [51] through actin filament reorganization and membrane blebbing [51, 52], conceivably providing a mechanism whereby MAPKs can mediate increased microglial proinflammatory cytokine release.

## 4. P2X_7_ Receptors and Neurological/Psychiatric Diseases

### 4.1. Neurodegenerative Disorders

P2X_7_ receptors may affect neuronal cell death through their ability to regulate the processing and release of IL-1*β*, a key mediator in neurodegeneration [53]. Deletion of the P2X_7_ receptor did not affect neuronal cell death induced by transient or permanent middle cerebral artery occlusion or by excitotoxic injury [54]. In another study, organotypic mouse hippocampal slice cultures were incubated for 3 hours to LPS, followed by a 3-hour coincubation with ATP or a P2X_7_ receptor agonist. A pronounced activation and apoptotic-like death of microglia was associated with a massive release of IL-1*β*, together with exacerbated CA3 pyramidal cell loss induced by subsequent exposure to the glutamatergic agonist *α*-amino-3-hydroxyl-5-methyl-4-isoxazole propionate in an IL-1*β*-dependent manner [55]. In rats subjected to spinal cord injury, areas surrounding the traumatic lesion displayed an abnormally high and sustained pattern of ATP release, and delivery of P2X_7_ antagonist after acute impact injury improved functional recovery and diminished cell death in the peritraumatic zone [56]. Acute spinal cord injuries produce highly inflammatory environments [11], and P2X_7_ receptor activation of local microglial cells may have adverse effects for neighboring neuronal cells. P2X_7_ may be involved in the generation of H_2_O_2_ in rat primary microglia [37, 57]. P2X_7_ receptor-like immunoreactivity was upregulated around *β*-amyloid plaques in Tg2576 mice (which overexpress the human amyloid precursor protein harboring the Swedish familial mutation (K670 → N, M671 → L)) and was regionally localized with activated microglia and astrocytes [37]. Upregulation of the P2X_7_ receptor subtype on microglia has been observed also after ischemia in the cerebral cortex of rats [58], and previous work has demonstrated immunoreactivity for the P2X_7_ receptor on reactive astrocytes in multiple sclerosis autopsy brain tissue [33].

**Figure 4:**
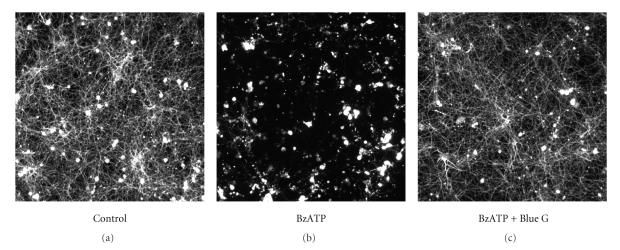
P2X_7_ receptor activation injures cortical neurons in vitro. Cocultures of rat cortical neurons and microglia were incubated for 3 days ± 100 *μ*M 2′, 3′-*O*-(4-benzoyl-benzoyl)ATP (BzATP) ± 3 *μ*M Brilliant Blue G (Blue G). Labeling for the neuron-specific marker *β*III-tubulin showed neurons to survive well and elaborate extensive neurite networks in cultures with unstimulated microglia (a), whereas BzATP caused a drastic and neuron-selective degeneration (b) that the P2X_7_ receptor antagonist Brilliant Blue G (c) prevented. Reproduced from Skaper et al. [57], with permission from Wiley-Liss, Inc.

Whether P2X_7_ receptor over-expression is driving microglial activation or, conversely, P2X_7_ receptor over-expression is a consequence of microglial activation is not known. Using cocultures of rat cortical neurons and microglia, Skaper et al. [57] have recently shown that ATP and BzATP cause neuronal cell injury. Treatment with the selective P2X_7_ antagonist Brilliant Blue G prevented the deleterious effects of BzATP-treated microglia (Figure 4). Neuronal cell injury was attenuated by a superoxide dismutase mimetic and by a peroxynitrite decomposition catalyst, suggesting a role for reactive oxide species [57]. Cocultures composed of wild-type cortical neurons and microglia from P2X_7_ receptor-deficient mice failed to exhibit neuronal cell injury in the presence of BzATP but retained sensitivity to injury when microglia were derived from genotypically matched normal (P2X_7_^+/+^ mice) [57]. P2X_7_ receptor activation on microglia thus appears necessary for microglial cell–mediated injury of neurons.

**Figure 5:**
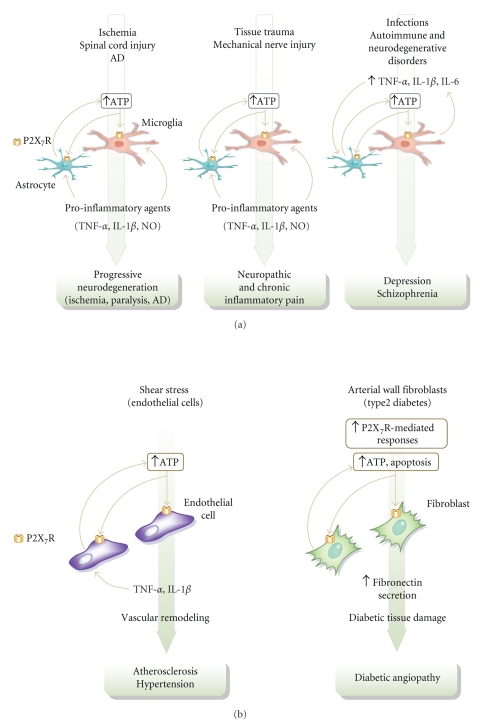
Schematic representation of the conditions which can lead to P2X_7_ receptor (P2X_7_R) activation in the nervous (a) and cardiovascular (b) systems. Tissue trauma, stress, mechanical injury, infection, and autoimmune disorders, among others, can lead to increased extracellular levels of ATP and/or proinflammatory cytokines. Extracellular ATP diffuses to activate neighboring cells by paracrine and autocrine pathways. In this context signaling through the P2X_7_ receptor may allow cells to sense and respond to events occurring in the extracellular environment, modulate the transcription of genes involved in cellular inflammatory processes, and thus regulate cytokine responses. The P2X_7_ receptor may function as an amplification device to spread the ATP wave as its activation triggers further ATP (and proinflammatory mediator) release, culminating in pathology. These characteristics, coupled with the broad distribution of P2X_7_ receptors encourage the therapeutic exploitation of this target. AD Alzheimer's disease.

A marked decline of intracellular ATP levels with a concomitant efflux of ATP into the extracellular space occurs in the rat brain during the first few minutes after oxygen depletion in vivo [59], and low concentrations of ATP can act as a chemoattractant for microglia [60], directing them to a site of injury. ATP released from activated astrocytes activates microglia [61], and microglial cells could encounter high levels of ATP near dying and disintegrating cells. These observations indicate that ATP and ATP analogues do act via the P2X_7_ receptor on microglia to affect neuronal cell health and that the P2X_7_ receptor can serve as an important component of a neuroinflammatory response (Figure 5(a)). Receptor antagonists of the P2X_7_ receptor could have therapeutic utility in the treatment of AD and cerebral ischemia and neuroinflammatory conditions by regulating pathologically activated glial cells.

### 4.2. Pain

ATP is recognized as one of the keys for the relay of sensory information from the periphery to the CNS [62], and is also one of several important mediators involved in immune-neural interactions [63]. Both sensory neurons and glial cells inside and outside of the CNS release ATP to affect surrounding cells [64, 65]. Particularly intriguing is the gathering body of literature linking activated microglia and astrocytes to central sensitization and the development and maintenance of neuropathic pain [65–67]. Both the localization of P2X_7_ receptors on pro-inflammatory cells, and the fact that ATP acting at P2X_7_ receptors serves as an efficient secondary stimulus for the generation and release of IL-1*β* from proinflammatory cells [68] have implicated a role for P2X_7_ receptors in inflammatory diseases [13] (Figure 5(a)).

Labasi et al. [35] observed a lower incidence and severity of monoclonal anticollagen-induced arthritis in P2X_7_ receptor knockout mice compared with wild-type, suggesting a pathological role for P2X_7_ receptors in inflammatory-/immune-mediated disease. Deletion of the *P*2*X*_7_ gene abolished the ability of ATP to induce IL-1*β* release from macrophages isolated from these mice [34]. Local administration of a P2X_7_ receptor antagonist had antihyperalgesic effects in the complete Freund's adjuvant-induced mechanical hyperalgesia (paw pressure) model [69]. More recently, Chessell et al. [36] demonstrated that in mice lacking the P2X_7_ receptor, inflammatory and neuropathic hypersensitivity is completely absent to both mechanical and thermal stimuli, while normal nociceptive processing is preserved. In these knockout animals, systemic cytokine analysis showed reductions in adjuvant-induced increases in IL-1*β*, IL-6, IL-10, and macrophage chemoattractant protein-1. Moreover, P2X_7_ receptor was upregulated in human dorsal root ganglia and injured nerves obtained from chronic neuropathic pain patients [36]. Endogenous IL-1 levels are increased in the nervous system in response to trauma associated with mechanical damage, ischemia, seizures, and hyperexcitability [70]. At the level of the spinal cord, blockage of IL-1 receptors results in reduced nociception in animal models of inflammation and nerve injury-induced pain [71, 72].

Much recent research has focused on the development of novel, selective, and potent small molecule inhibitors of the P2X_7_ receptor [73–77]. A-740003 and A-438079 are structurally unrelated P2X_7_ antagonists, and both exhibit therapeutic efficacy on neuropathy-induced mechanical allodynia [78, 79]. A-740003 also has antihyperalgesic effects in the carrageenan- and adjuvant-induced thermal hyperalgesia models of inflammatory pain [78]. These data are consistent with a study of an adamantane P2X_7_ antagonist (AACBA; GSK314181A) that is structurally dissimilar from A-740003 and A-438079, which showed dose-dependent antinociception in an inflammatory pain model [80]. The preclinical testing of P2X_7_ antagonists strongly suggests therapeutic potential in pathological pain and inflammation.

### 4.3. Depression

Intriguingly, cytokines like IL-1*β* are suggested to be involved in the pathophysiology of depression. This neuropsychiatric disorder is recognized as having high prevalence in several clinical settings including infectious, autoimmune, and neurodegenerative disorders, conditions associated with a proinflammatory status, and it has been proposed that excessive secretion of macrophage cytokines, for example, IL-1*β*, TNF-*α*, and IFN-*γ*, could be a potential causative factor [81]. Central and systemic administration of proinflammatory cytokines (IL-1*β*, TNF-*α*, IL-6) to animals induces what has been described as “sickness behavior,” which is characterized by many of the physiological and behavioral changes associated with depression [82, 83]. A similarity and functional linkage between symptoms of sickness behavior in animals and those of depression in humans has been suggested [83]. In addition, cytokines can induce neuroendocrine and neurochemical changes akin to a depressive syndrome [84], and clinical use of cytokines (e.g., IFN-*α*) produces depressive-like symptoms that can be attenuated with antidepressant treatment [85]. Not only do patients suffering from major depression, who are otherwise medically healthy, often have significant elevations in the density of microglia [86] and elevated levels of circulating proinflammatory cytokines [87–89] but also mice lacking functional type 1 or type 2 TNF-*α* receptors display an antidepressant phenotype [90]. Cytokines may thus be involved in the etiopathogenesis of depression (Figure 5(a)). 

Linkage studies have shown that the *P*2*X*_7_ gene may be involved in some neuropsychiatric conditions. Genetic analysis of a French population indicated a Gln640Arg single nucleotide polymorphism of the P2X_7_ receptor gene as a potential susceptibility gene for bipolar effective disorder [91] and major depression [92, 93]. This Gln640Arg polymorphism is located at the C-terminal domain of the P2X_7_ receptor, which is essential for its normal function. Identified polymorphisms in the P2X_7_ receptor of lymphocytes are known to produce a loss of function or to alter trafficking of the receptor to the membrane surface, thus decreasing its membrane expression [94]. The functional consequences for cytokine release of polymorphisms in the P2X_7_ receptor have been investigated in some cases, which result in reduction in TNF-*α* release from LPS stimulated leukocytes in the presence of ATP [95]. Basso et al. [96] have recently described the behavioral profile of P2X_7_ receptor gene knockout mice in animal models of depression and anxiety, and found an antidepressant-like phenotype together with a higher responsiveness to a subefficacious dose of the antidepressant imipramine. Further research will be necessary to elucidate the specific mechanism(s) underlying the antidepressant-like characteristics of P2X_7_ receptor knockout genotype and how inactivation of the *P*2*X*_7_ gene is physiologically translated into the expression of this behavioral profile.

Activation of the inflammatory response in the etiology of depression would lead one to predict that antidepressant drugs display negative immunoregulatory effects [97]. Indeed, a number of antidepressants that exhibit distinct mechanisms of action, at therapeutically effective concentrations, limit the release of proinflammatory cytokines both in vitro [98] and in vivo [99, 100]. In addition, antidepressants attenuate the behavioral and emotional disturbances elicited by immunostimulation and cytokine administration to humans and rodents [86, 100] and the abnormal increased production of proinflammatory cytokines seen in depressed patients [89, 101]. Antagonism of P2X_7_ receptors may thus constitute a novel target for the treatment of depression.

## 5. P2X_7_ Receptors and Cardiovascular Disease

ATP is an important neurotransmitter being released with noradrenaline and neuropeptide Y from perivascular sympathetic nerves; it acts at postjunctional P2X receptors to evoke vascular smooth muscle contraction. The relative contributions of ATP and noradrenaline as functional cotransmitters varies with species, age, type, and size of blood vessel, the tone/pressure of the blood vessel, and in disease [102]. In the vascular system, short-term purinergic signaling events are associated with the control of blood vessel tone/pressure influenced by ATP released from perivascular nerves, smooth muscle, and endothelial cells [102, 103]. In the rat vascular system, P2X_7_ receptor immunoreactivity was detected in all arteries, with the exception of small renal arteries [104]. In general, P2X_7_ receptor-specific immunoreactivity was seen in the outer adventitial layer with a predominantly vesicular distribution. In the large coronary and cerebral arteries, weak diffuse P2X_7_ receptor immunoreactivity was also detected in the smooth muscle layer [104]. P2X_7_ receptors are involved in sympathetic nerve-mediated vasoconstriction in small arteries of the rat hepatic mesentery [105]. Smooth muscle layers of placental and umbilical blood vessels express functional P2X_7_ receptors [106], suggesting their participation in the humoral regulation of placental blood flow. This is novel, since the umbilical cord lacks sympathetic innervation [107], a documented source of ATP. In addition, ATP is capable of increasing contractile tension in cardiac tissue via P2X receptors [108], although the receptor subtype was not identified. While ATP can also induce vasodilation in isolated aortic preparations, the nature of the purinergic receptor site responsible was not characterized [109–111]. 

Apoptotic cell death is recognized to occur in a number of vascular diseases, including atherosclerosis, restenosis, and hypertension [112, 113]. Vascular endothelial cells are continuously exposed to variations in blood flow, and the shear stress that occurs during changes in blood flow causes a substantial release of ATP from endothelial cells [114], which might mediate alterations in the balance between proliferation and apoptosis [115]. Occupancy of P2X_7_ receptors leads to the production of proinflammatory cytokines, and TNF-*α* promotes endothelial cell apoptosis via the activation of caspase 3 [113] which, conceivably, play a role in vascular remodeling in hypertension [116]. Stimulation of P2X_7_ receptors on human dendritic cells induces the release of tissue factor-bearing microparticles [117], which may have implications for triggering and propagating coagulation either in healthy or atherosclerotic vessels. P2X_7_ receptor activation reportedly amplifies LPS-induced vascular hyporeactivity, due to IL-1*β* release from endothelial cells, in turn inducing downstream nitric oxide production [118]. Thus, the P2X_7_ receptor may be an important regulator for vascular hypotensive responses in inflammation or inflammatory-related disease (Figure 5(b)). Intriguingly, evidence suggests that ambulatory blood pressure is associated with polymorphic variation in the P2X_7_ receptor gene [119].

In cutaneous vessels where purinergic neurotransmission is more prominent compared with deep vessels, physiological and pathological roles of nerve-released ATP have been described [120]. P2X_7_ receptors expressed in human saphenous vein myocytes contribute to the contractile effect of ATP [121], and venous diseases may offer conditions allowing P2X_7_ receptor activation to cause lysis of venous myocytes. ATP released after hypoxia, stress, and inflammation, or membrane damage, conditions found in the vessel wall of varicose veins [122], as well as that generated by reduced ecto-ATPase activity [123], may lead to P2X_7_ receptor-induced pore formation, the disorganization and loss of contractile myocytes in the muscle layers of the media of varicose veins, and venous disease.

It is well established that both ATP and noradrenaline are coreleased from sympathetic nerve varicosities [124]. Although in a range of muscular arteries both neurotransmitters contribute to neurally evoked contraction [125], ATP is the predominant sympathetic neurotransmitter in rat mesenteric arteries at high intraluminal pressure [126]. The increased responses produced by ATP at higher pressures could contribute to or exacerbate the raised pressure observed in hypertension. 

Fibroblasts are a key structural element of the arterial wall, major producers of extracellular matrix, and an active source of inflammatory mediators [127, 128]. In human pathology, fibroblast dysfunction is implicated in chronic degenerative diseases such as atherosclerosis and diabetic angiopathy [129]. In the atheromatous lesion, fibroblasts are a source of mediators that stimulate endothelial cells and promote recruitment of leukocytes, thus accelerating damage of the arterial intima and media [127]. In diabetes, the arterial wall undergoes accelerated degenerative changes [130], the pathogenesis of which is incompletely understood but that undoubtedly implicates profound modifications of fibroblast reactivity. In diabetic patients, fibroblast responses might be inherently aberrant [131], thus rendering these cells sensitive to inflammatory factors released into the blood or the arterial wall. It is likely that ATP is released at the site of atherosclerotic lesions or during platelet adhesion to the endothelium [132]. It is interesting to note a recent study demonstrating that fibroblasts from type-2 diabetes patients are characterized by a hyperactive purinergic loop based either on a higher level of ATP release or an enhanced P2X_7_ receptor reactivity, together with an increased pericellular concentration of ATP, and a higher basal level of fibronectin secretion and spontaneous rate of apoptosis at least in part dependent on autocrine stimulation of P2X_7_ receptors by secreted ATP [133] (Figure 5(b)). Accumulation of fibronectin in the interstitial space (e.g., arterial wall) in diabetes is believed to play a major role in the pathogenesis of diabetic tissue damage [134]. In another report, P2X_7_ receptor activation in diabetic rabbits led to a marked reduction in retinal blood velocity and function [135]. 

## 6. Concluding Remarks

It is now generally accepted that high levels of extracellular nucleotides such as ATP may be released under pathological conditions such as inflammation, trauma, and stress. Interestingly, a number of neurodegenerative conditions exhibit enhanced P2X_7_ receptor expression in the neuroinflammatory loci where activated microglia are a coexisting feature. Recent findings suggest that increased P2X_7_ receptor numbers drive microglial activation, rather than P2X_7_ receptor over-expression being a consequence of microglial activation [136]. Signaling via P2X_7_ receptors may thus allow cells to sense and respond to events occurring in the extracellular environment, modulate the transcription of genes involved in cellular inflammatory processes, and to thus regulate cytokine responses. Given the distribution of P2X_7_ receptors and the fact that high concentrations of ATP are required to activate the receptor, this P2X receptor may be viewed as a ‘danger’ sensor. The therapeutic exploitation of P2X_7_ receptors is now under way because of their potential role, not only in such disorders as AD, spinal cord injury, and sensory neuropathies [137] but also in multiple sclerosis [138], inflammatory neuropathic pain [36], rheumatoid arthritis [35], as well as depressive illness. The discovery of P2X_7_ receptor-selective antagonists has provided data demonstrating that the acute blockage of P2X_7_ receptors significantly reduces nociception in animal models of persistent neuropathic and inflammatory pain, while there is growing appreciation for the role of P2X_7_ receptor modulation of proinflammatory IL-1*β* processing [139], the analgesic activity of P2X_7_ receptor antagonists [78, 79] indicates a specific role for P2X_7_ receptors in neuronal-glial cell interactions associated with ongoing pain [23]. P2X_7_ receptors are expressed with some selectivity on different types of cells in the cardiovascular system, and drugs affecting P2X_7_ receptor signaling may have promise as clinical antihypertensive and antithrombotic agents [140]. In this context, the P2X_7_ receptor may be viewed as a key point of communication between the nervous, immune, and cardiovascular systems. Further investigation of the P2X_7_ receptor with receptor subselective antagonists in preclinical studies as well as in disease-specific clinical trials will help to evaluate this target's potential therapeutic use.
